# Diatom Biodiversity and Speciation Revealed by Comparative Analysis of Mitochondrial Genomes

**DOI:** 10.3389/fpls.2022.749982

**Published:** 2022-03-24

**Authors:** Yichao Wang, Shuya Liu, Jing Wang, Yanxin Yao, Yang Chen, Qing Xu, Zengxia Zhao, Nansheng Chen

**Affiliations:** ^1^Chinese Academy of Sciences Key Laboratory of Marine Ecology and Environmental Sciences, Institute of Oceanology, Chinese Academy of Sciences, Qingdao, China; ^2^Laboratory of Marine Ecology and Environmental Science, Qingdao National Laboratory for Marine Science and Technology, Qingdao, China; ^3^College of Planetary and Earth Sciences, University of Chinese Academy of Sciences, Beijing, China; ^4^Center for Ocean Mega-Science, Chinese Academy of Sciences, Qingdao, China; ^5^College of Life Science and Technology, Huazhong Agricultural University, Wuhan, China; ^6^Jiaozhou Bay National Marine Ecosystem Research Station, Institute of Oceanology, Chinese Academy of Sciences, Qingdao, China; ^7^Department of Molecular Biology and Biochemistry, Simon Fraser University, Burnaby, BC, Canada

**Keywords:** diatom, mitochondrial genome, comparative genomics, evolution, diversity, divergence time

## Abstract

Diatoms (Bacillariophyta) constitute one of the most diverse and ecologically significant groups of phytoplankton, comprising 100,000–200,000 species in three classes Bacillariophyceae, Mediophyceae, and Coscinodiscophyceae. However, due to the limited resolution of common molecular markers including 18S rDNA, 28S rDNA, ITS, *rbcL*, and *cox1*, diatom biodiversity has not been adequately ascertained. Organelle genomes including mitochondrial genomes (mtDNAs) have been proposed to be “super barcodes” for distinguishing diatom species because of their rich genomic content, and the rapid progress of DNA sequencing technologies that has made it possible to construct mtDNAs with increasing throughout and decreasing cost. Here, we constructed complete mtDNAs of 15 diatom species including five Coscinodiscophyceae species (*Guinardia delicatula*, *Guinardia striata*, *Stephanopyxis turris*, *Paralia sulcata*, and *Actinocyclus* sp.), four Mediophyceae species (*Hemiaulus sinensis*, *Odontella aurita var. minima*, *Lithodesmioides* sp., and *Helicotheca tamesis*), and six Bacillariophyceae species (*Nitzschia ovalis*, *Nitzschia* sp., *Nitzschia traheaformis*, *Cylindrotheca closterium*, *Haslea tsukamotoi*, and *Pleurosigma* sp.) to test the practicality of using mtDNAs as super barcodes. We found that mtDNAs have much higher resolution compared to common molecular markers as expected. Comparative analysis of mtDNAs also suggested that mtDNAs are valuable in evolutionary studies by revealing extensive genome rearrangement events with gene duplications, gene losses, and gains and losses of introns. Synteny analyses of mtDNAs uncovered high conservation among species within an order, but extensive rearrangements including translocations and/or inversions between species of different orders within a class. Duplication of *cox1* was discovered for the first time in diatoms in *Nitzschia traheaformis* and *Haslea tsukamotoi*. Molecular dating analysis revealed that the three diatom classes split 100 Mya and many diatom species appeared since 50 Mya. In conclusion, more diatom mtDNAs representing different orders will play great dividends to explore biodiversity and speciation of diatoms in different ecological regions.

## Introduction

Diatoms (Bacillariophyta) constitute a major group of phytoplankton as free living organisms in both marine and freshwater environments or as endosymbionts in dinoflagellates and foraminifers (Field et al., 1998; Pierella Karlusich et al., 2020). Diatoms are unicellular photosynthetic microalgae deemed to be of crucial significance in biogeochemical cycles and the functioning of aquatic food webs (Smetacek, 1998; Armbrust, 2009; Falciatore et al., 2020). Diatoms have the most effective RuBisCO enzyme within autotrophs and work as an essential part in the cycling of CO_2_, which is predicted to be comparable to that of all terrestrial rainforests combined in global carbon cycling (Field et al., 1998; Giordano et al., 2005) and constitute a large part of aquatic biomass contributing approximately 20% of the total primary production on Earth (Nelson et al., 1995; Falkowski et al., 1998). Diatoms also have great value in commercial applications. Combination of different diatom species provides better balanced nutrition and improves aquaculture animal growth better such as *Chaetoceros*, *Skeletonema*, and *Thalassiosira* (Spolaore et al., 2006). *Odontella aurita* is a marine diatom with rich eicosapentaenoic acid (EPA) content and displays beneficial effect in reducing risk factors for high-fat induced metabolic syndrome (Haimeur et al., 2012). The high neutral lipid content and growth rate of *Fistulifera solaris* are beneficial for biodiesel production (Tanaka et al., 2015). Diatom species can also pose substantial negative impact on environment. Extreme successions of some diatom species under certain situation can cause harmful algal blooms (HABs) with serious negative consequences on human health, as well as economic loss and social disruption (Jin et al., 2008; Dyson and Huppert, 2010; Lelong et al., 2012; Crosman et al., 2019; Moore et al., 2020). For example, *Pseudo-nitzschia pungens* has been found to cause HABs in coastal regions of New Zealand and the west coastal regions of America (Patrick et al., 2016).

The critical importance of diatoms is to some degree dictated by their biodiversity. Diatoms are divided into three classes Bacillariophyceae, Mediophyceae, and Coscinodiscophyceae (Medlin and Kaczmarska, 2019). Diatom species show high variations in cell sizes, ranging from a few micrometers to a few millimeters, with single cells forming chains of connected cells for many species (Foster et al., 2011). While morphological features have been effectively used as defining criteria for taxonomy of the diatom species, taxonomy for diatom species with small-cell sizes, and few discrete morphological characters under light microscope are challenging (Mann et al., 2021). Many molecular markers including 18S rDNA, 28S rDNA, ITS, *rbcL*, and *cox1* have been successfully applied alone or in combination with microscopy to study diatom species (Evans et al., 2007; Hamsher et al., 2013; Park et al., 2017). While these common molecular markers are normally effective for distinguishing higher taxa, they are often inadequate for distinguishing congener species, let alone intra-species genetic diversity (Wilcox, 1998; Hollingsworth et al., 2011; Song et al., 2020). As such, although between 100,000 and 200,000 diatom species have been estimated to exist (Malviya et al., 2016), currently only about 12,000 species have been identified (Mann and Vanormelingen, 2013). Lack of molecular markers with sufficient resolution impedes in-depth analysis of diatom biodiversity and speciation, and particularly their differential contribution to ecological systems.

With the development of DNA sequencing technologies and bioinformatics analysis algorithms, mitochondrial genomes (mtDNAs) of thousands species have been fully constructed, which helped to uncover valuable knowledge about phylogenetic relationships and evolutionary trajectories (Smith, 2016). The research of diatom mtDNAs began with the construction of the mtDNA (a circular DNA of 43,827 bp) of *Thalassiosira pseudonana* (Armbrust et al., 2004). Subsequent construction of the *Phaeodactylum tricornutum* mtDNA revealed a 35,454-bp repeat sequence, providing a first glimpse of rich divergence among diatoms mtDNAs (Secq and Green, 2011). Further analysis of mtDNAs uncovered the existence of group I intron, encoding a LAGLIDADG endonuclease in the mtDNA of the endosymbiont diatom *Kryptoperidinium foliaceum* (Imanian et al., 2012). Comparative analysis of five mtDNAs (*Toxarium undulatum*, *Psammoneis japonica*, *Eunotia naegelii*, *Cylindrotheca closterium*, and *Nitzschia* sp.) with eleven publicly available diatom mtDNAs suggested that diatom mtDNAs shared strongly conserved genes and highly variable intron content caused by intron loss and horizontal transfer (Guillory et al., 2018). The mtDNAs have also been used as “super barcodes” with higher resolution than common molecular marker genes for comparative genomics analysis (Lv et al., 2020). Mitochondrial genomes have been used to track the genetic diversity of the harmful algal species *Eucampia zodiacus* (Zhang W. et al., 2021). Despite their critical importance, mtDNAs of only 53 diatom species of seventeen orders have been successfully constructed, with many orders underrepresented. In particular, only four mtDNAs have been constructed for species in the class Coscinodiscophyceae. Such limited availability of mtDNAs hinders in-depth research on diatom biodiversity and the speciation of diatoms.

The Jiaozhou Bay, which is connected to the Yellow Sea with a small opening, is an epitome of China’s offshore ecosystem (Liu et al., 2004). Previous studies revealed rich diatom composition in the Jiaozhou Bay, which makes it suitable for studying the biodiversity and speciation of diatom (Liu and Chen, 2021). In this study, we constructed complete mtDNAs of 15 diatom species, which was the first step in constructing mtDNAs for all diatom species in the Jiaozhou Bay. All of these 15 strains were isolated from the Jiaozhou Bay, consisting of strains from 10 diatom orders including two orders Bacillariales (four species) and Naviculales (two species) of the class Bacillariophyceae, four orders Eupodiscales (one species), Hemiaulales (one species), Briggerales (one species) and Lithodesmiales (one species) of the class Mediophyceae, and four orders from Paraliales (one species), Stephanopyxales (one species), Rhizosoleniales (two species), and Coscinodiscales (one species) of the class Coscinodiscophyceae. For accurate comparison, we re-annotated 53 published mtDNAs publicly available at GenBank at NCBI and made several annotation corrections. This work enriched diatoms’ mtDNA resources for comparative analysis of biodiversity and speciation, and represents the first large-scale construction of mtDNAs for diatom species from the Jiaozhou Bay.

## Materials and Methods

### Strain Isolation and Culturing

**FIGURE 1:**
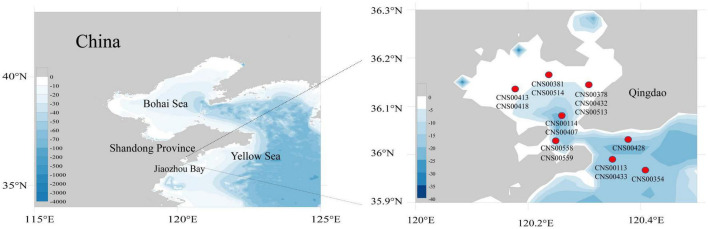
Sampling sites of 15 diatom species in the Jiaozhou Bay.

Fifteen diatom strains (CNS00113, CNS00114, CNS00354, CNS00378, CNS00381, CNS00407, CNS00413, CNS00418, CNS00428, CNS00432, CNS00433, CNS00513, CNS00514, CNS00558, and CNS00559) were isolated from water samples collected in the Jiaozhou Bay (Figure 1), onboard the R/V Chuangxin, which was operated by The Jiaozhou Bay Marine Ecosystem Research Station using single-cell capillary methods. Cells were cultured in L1 medium with 1‰ volume fraction Na_2_SiO_3_ with H_2_O added. The culture temperature was 18–20°C, and the illuminance was from 2,000 to 3,000 Lx at the photoperiod of 12 h light–12 h dark.

### DNA Library Preparation and Whole Genome Sequencing

Total DNA was extracted with DNAsecure Plant Kit (Tiangen Biotech, Beijing, China). The genomic DNA samples were fragmented by sonication (Covaris S220, Covaris, WBN, United States) to a size of 350 bp. DNA fragments were then end polished, A-tailed, and ligated with the full-length adapters for Illumina sequencing, followed by PCR (MiniAmp Thermal Cycler, ThermoFisher, MA, United States) enrichment using generic adapter P5 and P7 oligos. The DNA libraries were sequenced using NovaSeq PE150 (Illumina, San Diego, CA, United States) and paired-end reads in size of 150 bp were generated.

### Morphological and Molecular Identification of Diatom Strains

**FIGURE 2:**
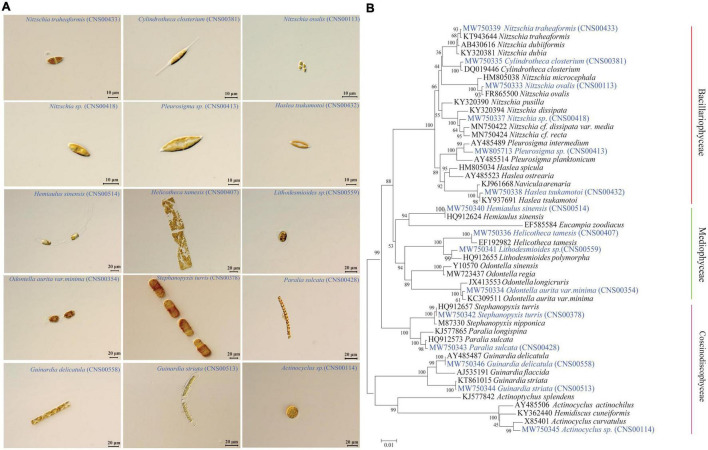
Morphological and molecular anlaysis of diatom species analyzed in this study. **(A)** Representative micrographs of 15 diatom species studied in this project. **(B)** Phylogenetic analysis based on 18S ribosomal DNA (18S rDNA) gene. Numbers at the branches represent bootstrap values. Branch lengths are proportional to the genetic distances, which are indicated by the scale bar.

All diatom strains were observed using microscope (Zeiss Axio Imager Z2) (Figure 2A). Common molecular marker 18S rDNA for each strain were constructed using whole genome sequencing results. The phylogenetic trees of 18S rDNA were constructed using MEGA7 (Kumar et al., 2016). Phylogenetic relationships were inferred using the Neighbor-Joining method (Saitou and Nei, 1987). The percentage of replicate trees in which the associated taxa clustered together in the bootstrap test (1,000 replicates) was shown next to the branches (Felsenstein, 1985). The analyses involved 50 nucleotide sequences in 18S rDNA gene. All positions containing gaps and missing data were eliminated. There were a total of 1,518 positions in the final dataset of 18S rDNA gene.

### Construction and Annotation of Mitochondrial Genomes

Raw data was trimmed using Trimmomatic-0.39 with the parameters: LEADING:3 TRAILING:3 SLIDINGWINDOW:4:15 MINLEN:75 (Bolger et al., 2014). Clean reads were assembled to get complete mtDNAs using GetOrganelle-1.7.4.1 (Jin et al., 2020). The mtDNA sequences were validated using BWA-0.7.17 (Li and Durbin, 2009), SAMtools-1.10 (Li et al., 2009), and IGV-2.7.2 (Thorvaldsdottir et al., 2013). Annotations were conducted with MFannot^1^ and NCBI’s ORF Finder,^2^ completed in NCBI’s Sequin15.10^3^ with genetic code of Mold, Protozoan, and Coelenterate Mitochondrial; Mycoplasma/Spiroplasma. For the accuracy of comparative analysis, we had inspected and re-annotated the 53 mtDNAs which were downloaded from the NCBI.

### Phylogenetic Analysis of Mitochondrial Genomes

The amino acid sequences encoded by 27 mitochondrial protein-coding genes (*atp6, 8, 9; cob; cox1, 2, 3; nad1-7, 4L, 9, 11; rpl2, 6, 14, 16; rps3, 4, 8, 11, 14;* and *tatC*) from each diatom species were extracted and concatenated for phylogenetic analysis and *Phytophthora ramorum* in Oomycota was used as outgroup according to previous studies (Martin, 2008; An et al., 2017; Liu et al., 2019a). The amino acid sequences encoded by the 27 genes were individually aligned using MAFFT (Katoh and Standley, 2013). Ambiguously aligned regions were trimmed with using trimAl 1.2rev59 (Capella-Gutierrez et al., 2009), and then concatenated using Phyutility (Smith and Dunn, 2008). The best-fit model was found by ModelFinder (Kalyaanamoorthy et al., 2017). The incongruence length difference test (ILD, also called the partition homogeneity test) was conducted via PAUP4.0 with the following parameters: number of replicates = 100; optimality criterion = maximum parsimony (Swofford, 2002). The *p*-value (0.01) indicated that combining data would not affect the phylogenetic accuracy. When the ILD detects *p* values lower than 0.001, the combined data performs less well than the individual partitions (Cunningham, 1997). The phylogenetic tree was constructed with IQ-TREE (Trifinopoulos et al., 2016). The ultrafast bootstrap analysis with 1,000 replicates of the dataset and approximate Bayes test were conducted to estimate statistical reliability (Anisimova et al., 2011; Bui Quang et al., 2013). The datasets used for phylogenetic inference are provided in Supplementary Material.

### Synteny Analysis

Synteny analysis of mtDNA sequences was carried out with the program Mauve v2.3.1 using progressiveMauve (Darling et al., 2010). The comparative illustration of mtDNAs was performed using circos-0.69 (Krzywinski et al., 2009).

### Divergence Time Estimation

Phylogenetic analysis and molecular dating were analyzed by calculating the codon evolution rate of 27 mitochondrial protein-coding gene nucleotide sequences (*atp6, 8, 9; cob; cox1, 2, 3; nad1-7, 4L, 9, 11; rpl2, 6, 14, 16; rps3, 4, 8, 11, 14; and tatC*). The *Ectocarpus siliculosus was* used as outgroup with its fossil age (Supplementary Table 1). The nucleotide sequence of 27 protein-coding genes (PCGs) were aligned using MAFFT with Codon and concatenated in the software PhyloSuite (Zhang et al., 2020). The phylogenetic tree was performed with IQ-TREE. Molecular dating was conducted using PAML package (Yang, 2007). Briefly, rough estimation of substitution rate was conducted using baseml, and estimation of divergence times with the approximate likelihood method was performed using mcmctree. Five calibration points were used in this analysis (Supplementary Table 1). The phylogenetic tree was displayed in the Figtree and visualized with 95% highest posterior density interval (HPD) for each node.

## Results

### Morphological and Molecular Characterization of 15 Species

Fifteen diatom strains analyzed in this study were all isolated from the Jiaozhou Bay, China (Figure 1). These strains were annotated based on their morphological features (Figure 2A) and the similarity of the molecular marker 18S rDNA sequence of each strain to those of known species (Figure 2B), with percentage identity (PID) ≥ 99% to corresponding reference sequences. Three strains CNS00433, CNS00113, and CNS00418, which were unicellular cells that were long, straight, and ovoid, shared morphologies that resembled *Nitzschia* species (Trobajo et al., 2013). The strain CNS00433 was annotated as *Nitzschia traheaformis* because its full-length 18S rDNA clustered closely with that of *Nitzschia traheaformis* in the phylogenetic tree (Figure 2B) with high PID (99.69% with KT943644) (Table 1) (Witkowski et al., 2016). The strain CNS00113 was annotated as *Nitzschia ovalis* (PID = 99.82% with FR865500) (Gachon et al., 2013), while the strain CNS00418 was annotated as *Nitzschia* sp. (PID = 98.70% with KY320394). The strain CNS00381, whose cells were unicellular and long with cylindrical central part and elongated ends, was annotated as *Cylindrotheca closterium* (99.71% with DQ019446) (Williams, 2007). The cells of strains CNS00413 and CNS00432 were all spindle-like. The strain CNS00413 was annotated as *Pleurosigma* sp. (98.31% PID with AY485514) (Damste et al., 2004), while the strain CNS00432, which had a “sandwich-type” valve structure, was annotated as *Haslea tsukamotoi* (99.61% PID with KY937691) (Li et al., 2017). The strain CNS00514, whose cells had protrusions at the ends that connected the cells into a curved chain, was annotated as *Hemiaulus sinensis* (100% PID with HQ912624) (Theriot et al., 2010). The strain CNS00407, whose flat cells were tightly connected and twisted into a chain, was annotated as *Helicotheca tamesis* (99.64% PID with EF192982) (Rampen et al., 2007). The strain CNS00559, whose cells were irregular polygon and possessed numerous centrally located plastids, was annotated as *Lithodesmioides* sp. (98.80% PID with HQ912655) (Theriot et al., 2010). The strain CNS00354, whose cells had long thorns in the middle was annotated as *Odontella aurita var. minima* (99.63% PID with KC309511) (Ashworth et al., 2013). The strains CNS00378, CNS00428, CNS00558, CNS00513, and CNS00114 were annotated as species in the class Coscinodiscophyceae based on their morphological features (Figure 2A). The strains CNS00378, CNS00558, and CNS00513 were annotated as *Stephanopyxis turris* (99.94% PID with HQ912657), *Guinardia delicatula* (99.94% PID with AY485487), and *Guinardia striata* (99.94% PID with KT861015), respectively (Damste et al., 2004; Theriot et al., 2010). The strain CNS00428 was annotated as *Paralia sulcata* (99.71% PID with HQ912573) (Theriot et al., 2010). The strain CNS00114, whose cells were round did not form chain, was annotated as *Actinocyclus* sp. (98.86% PID with X85401) (Kooistra and Medlin, 1996).

**TABLE 1:** 18S rDNA information of 15 strains from the Phylum Bacillariophyta.

Strains	Species	18S rDNA Accession number	Accession number of references	Percent identity(%)
CNS00113	*Nitzschia ovalis*	MW750333	FR865500	99.82
CNS00418	*Nitzschia* sp.	MN750337	KY320394	98.70
CNS00433	*Nitzschia traheaformis*	MW750339	KT943644	99.69
CNS00381	*Cylindrotheca closterium*	MW750335	DQ019446	99.71
CNS00432	*Haslea tsukamotoi*	MW750338	KY937691	99.61
CNS00413	*Pleurosigma* sp.	MW805713	AY485489	98.31
CNS00514	*Hemiaulus sinensis*	MW750340	HQ912624	100.00
CNS00354	*Odontella aurita var.minima*	MW750334	KC309511	99.63
CNS00559	*Lithodesmioides* sp.	MW750341	HQ912655	98.80
CNS00407	*Helicotheca tamesis*	MW750336	EF192982	99.64
CNS00558	*Guinardia delicatula*	MW750346	AY485487	99.94
CNS00513	*Guinardia striata*	MW750344	KT861015	99.63
CNS00378	*Stephanopyxis turris*	MW750342	HQ912657	99.63
CNS00428	*Paralia sulcata*	MW750343	HQ912573	99.71
CNS00114	*Actinocyclus* sp.	MW750345	X85401	98.86

### Construction and Comparative Analysis of Mitochondrial Genomes

**FIGURE 3:**
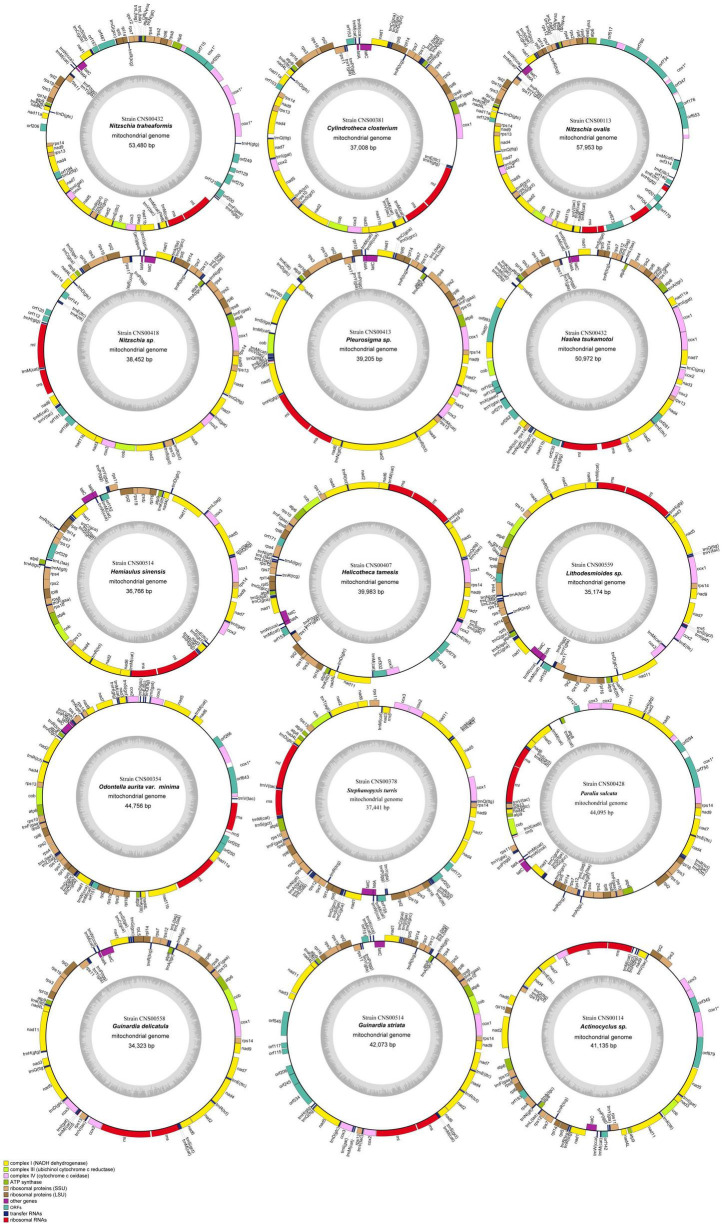
Circular maps of 15 complete mtDNAs. Genes shown on the inside of the map are transcribed in a clockwise direction, whereas those on the outside of the map are transcribed counterclockwise. The assignment of genes into different functional groups is indicated by colors. The ring of bar graphs on the inner circle shows the AT content in light gray.

We successfully constructed the complete mtDNA for all 15 diatom strains, among which 14 mtDNAs representing 14 species were constructed for the first time (Figure 3). The mtDNA of *Cylindrotheca closterium*, whose mtDNA was previously published, was constructed for a second strain collected from Jiaozhou Bay for comparative analysis. The sizes of these 15 mtDNAs varied substantially, ranging from 34,323 bp (*Guinardia delicatula*, order Rhizosoleniales, class Coscinodiscophyceae) to 57,953 bp (*Nitzschia ovalis*, order Bacillariales, class Bacillariophyceae) (Table 2). The AT contents also varied substantially, ranging from 68.0% (*Nitzschia ovalis*) to 77.8% (*Actinocyclus* sp.). Interestingly, of these mtDNAs, genome sizes and AT contents showed a significant negative correlation (Pearson correlation coefficient = −0.59, *p* < 0.05), suggesting that mtDNAs with large sizes tend to have relatively lower AT contents. Further study on more mtDNAs is needed to ascertain the relationship between AT contents and mtDNA genome sizes.

**TABLE 2:** Genome features of 15 mtDNA from the Phylum Bacillariophyta.

Class	Order	Species	Strain	Accession number	Size (bp)	A+T (%)
Bacillariophyceae (6)	Bacillariales	*Nitzschia ovalis*	CNS00113	MW849263	57953	68.0
	Bacillariales	*Nitzschia* sp.	CNS00418	MW849267	38452	72.8
	Bacillariales	*Nitzschia traheaformis*	CNS00433	MW849269	53480	70.1
	Bacillariales	*Cylindrotheca closterium*	CNS00381	MW849265	37008	69.1
	Naviculales	*Haslea tsukamotoi*	CNS00432	MW849268	50972	71.4
	Naviculales	*Pleurosigma* sp.	CNS00413	MW861541	39205	75.0
Mediophyceae (4)	Hemiaulales	*Hemiaulus sinensis*	CNS00514	MW849270	36766	73.1
	Eupodiscales	*Odontella aurita var.minima*	CNS00354	MW849264	44756	70.6
	Lithodesmiales	*Lithodesmioides* sp.	CNS00559	MW849271	35174	74.8
	Briggerales	*Helicotheca tamesis*	CNS00407	MW849266	39983	74.1
Coscinodiscophyceae (5)	Rhizosoleniales	*Guinardia delicatula*	CNS00558	MW413905	34323	73.9
	Rhizosoleniales	*Guinardia striata*	CNS00513	MW413904	42073	71.8
	Stephanopyxales	*Stephanopyxis turris*	CNS00378	MW413903	37441	74.3
	Paraliales	*Paralia sulcata*	CNS00428	MW413901	44095	73.9
	Coscinodiscales	*Actinocyclus* sp.	CNS00114	MW413902	41135	77.8

**FIGURE 4:**
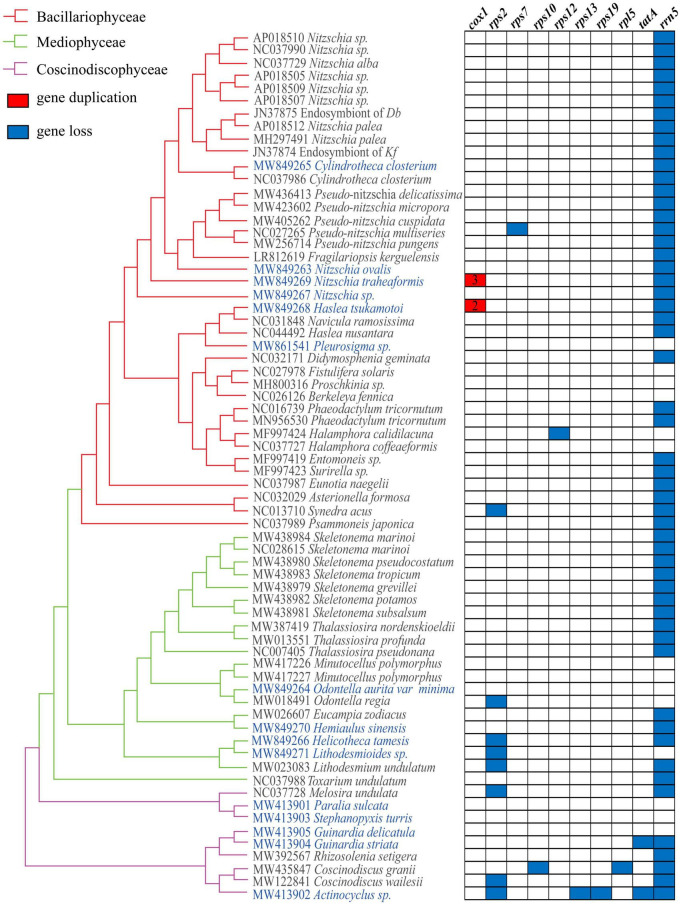
Gene losses and gains in diatom mtDNAs. The matrix shows 10 genes variably present among 68 mtDNAs.

With the 15 mtDNAs constructed in this study, there are altogether 68 mtDNA constructed for all Bacillariophyta species. Of these 68 mtDNAs, 39 mtDNAs were for species in Bacillariophyceae, 20 mtDNAs were for species in Mediophyceae, while only nine mtDNAs were for species in Coscinodiscophyceae (Table 3). Most mtDNAs contained 35 PCGs and two non-coding rRNA genes (*rns* and *rnl*). The mtDNAs PCGs of *Nitzschia traheaformis* and *Haslea tsukamotoi* were exceptional because they possessed three and two copies of *cox1*, respectively (Figure 4). Different copies of *cox1* genes had different lengths, suggesting prolonged evolution after the duplication events. The numbers of tRNA genes varied from 20 (*Actinocyclus* sp.) to 28 (*Psammoneis japonica*). Most instances of introns were found in *cox1* and *rnl* (Table 3). However, introns were also found in other genes including *nad5* and *nad11* in the mtDNAs of *Haslea tsukamotoi* and *Pleurosigma* sp., in *cox2*, *cox3*, *cob*, *nad7*, and *rns* in *Halamphora calidilacuna* mtDNA, and *nad2* in *Eunotia naegelii* mtDNA. Among these introns, only four group I introns were found, including one found in *cox1* in the mtDNAs of *Nitzschia ovalis*, one found in *rns* in the mtDNA of *Halamphora calidilacuna*, and two found in *cox1* and *rnl* in the mtDNA of Endosymbiont of *Kryptoperidinium foliaceum* (Table 3).

**TABLE 3:** Mitochondrial gene content of 68 mtDNAs from the Phylum Bacillariophyta.

	Species	Accession number	PCGs/rRNAs	tRNA	Introns (I/II)	Location of introns (I/II)
Bacillariophyceae (39)	*Nitzschia ovalis*	MW849263	35/2	26	1/5	*cox1***/***cox1*; *rnl*
	*Nitzschia* sp.	MW849267	35/2	25	0	-
	*Nitzschia traheaformis*	MW849269	37/2	25	0/1	-/*cox1*
	*Cylindrotheca closterium*	MW849265	35/2	24	0	-
	*Haslea tsukamotoi*	MW849268	36/2	25	0/1	-/*nad5*
	*Pleurosigma* sp.	MW861541	35/3	25	0/1	-/*nad11*
	*Psammoneis japonica*	NC_037989	35/2	28	0/11	-/*cox1*; *rnl*
	*Cylindrotheca closterium*	NC_037986	35/2	24	0/1	-/*cox1*
	*Fragilariopsis kerguelensis*	LR812619	35/2	24	0	-
	*Nitzschia palea*	MH297491	35/2	24	0	-
	*Nitzschia palea*	AP018512	35/2	24	0	-
	*Nitzschia alba*	NC_037729	35/2	24	0	-
	*Nitzschia* sp.	AP018507	35/2	24	0	-
	*Nitzschia* sp.	AP018509	35/2	24	0	-
	*Nitzschia* sp.	NC_037990	35/2	24	0	-
	*Nitzschia* sp.	AP018510	35/2	24	0	-
	*Nitzschia* sp.	AP018505	35/2	24	0	-
	*Pseudo-nitzschia multiseries*	NC_027265	34/2	24	0/2	-/*cox1*
	*Pseudo-nitzschia delicatissima*	MW436413	35/2	24	0/1	-/*cox1*
	*Pseudo-nitzschia micropora*	MW423602	35/2	24	0	-
	*Pseudo-nitzschia cuspidata*	MW405262	35/2	24	0	-
	*Pseudo-nitzschia pungens*	MW256714	35/2	24	0/1	-/*cox1*
	*Didymosphenia geminata*	NC_032171	35/2	25	0	-
	*Entomoneis* sp.	MF997419	35/2	23	0	-
	*Halamphora calidilacuna*	MF997424	34/3	26	1/19	*rns/cox1*,*2*,*3*;*nad7*;*cob*; *rnl*,*rns*
	*Halamphora coffeaeformis*	NC_037727	35/3	24	0/5	-/*cox1*; *rnl*
	*Berkeleya fennica*	NC_026126	35/3	25	0	-
	*Fistulifera solaris*	NC_027978	35/3	24	0	-
	*Haslea nusantara*	NC_044492	35/2	24	0	-
	*Navicula ramosissima*	NC_031848	35/3	23	0/5	-/*cox1*
	*Phaeodactylum tricornutum*	MN956530	35/2	24	0/4	-/*cox1;rnl;rns*
	*Phaeodactylum tricornutum*	NC_016739	35/2	23	0/4	-/*cox1;rnl;rns*
	*Proschkinia* sp.	MH800316	35/3	24	0/4	-/*cox1*; *rnl*
	*Surirella* sp.	MF997423	35/2	22	0	-
	*Endosymbiont of Kryptoperidinium foliaceum*	JN378734	35/2	22	2/1	*cox1*; *rnl/cox1*; *rnl*
	*Endosymbiont of Durinskia baltica*	JN378735	35/2	23	0	-
	*Eunotia naegelii*	NC_037987	35/2	23	0/2	-/*rnl*; *nad2*
	*Asterionella formosa*	NC_032029	35/2	24	0/1	-/*cox1*
	*Synedra acus (Ulnaria acus)*	NC_013710	34/2	24	0/3	-/*cox1*; *rnl*
Mediophyceae (20)	*Hemiaulus sinensis*	MW849270	35/2	24	0	-
	*Odontella aurita var.minima*	MW849264	35/3	24	0/1	-/*cox1*
	*Lithodesmioides* sp.	MW849271	34/3	24	0	-
	*Helicotheca tamesis*	MW849266	34/2	24	0	-
	*Odontella regia*	MW018491	34/3	24	0	-
	*Lithodesmium undulatum*	MW023083	34/3	25	0	-
	*Minutocellus polymorphus*	MW417226	35/3	23	0	-
	*Minutocellus polymorphus*	MW417227	35/3	23	0	-
	*Eucampia zodiacus*	MW026607	35/2	24	0	-
	*Thalassiosira pseudonana*	NC_007405	35/2	25	0/1	-/*cox1*
	*Thalassiosira nordenskioeldii*	MW387419	35/2	27	0	-
	*Thalassiosira profunda*	MW013551	35/2	25	0	-
	*Skeletonema marinoi*	NC_028615	35/2	25	0	-
	*Skeletonema marinoi*	MW438984	35/2	25	0	-
	*Skeletonema tropicum*	MW438983	35/2	25	0	-
	*Skeletonema potamos*	MW438982	35/2	26	0	-
	*Skeletonema subsalsum*	MW438981	35/2	26	0	-
	*Skeletonema pseudocostatum*	MW438980	35/2	25	0	-
	*Skeletonema grevillei*	MW438979	35/2	25	0	-
	*Toxarium undulatum*	NC_037988	35/2	26	0	-
Coscinodiscophyceae (9)	*Guinardia delicatula*	MW413905	35/3	24	0	-
	*Guinardia striata*	MW413904	34/2	24	0	-
	*Stephanopyxis turris*	MW413903	35/3	25	0	-
	*Paralia sulcata*	MW413901	35/3	26	0/1	-/*cox1*
	*Actinocyclus* sp.	MW413902	31/2	20	0/1	-/*cox1*
	*Rhizosolenia setigera*	MW392567	35/2	24	0	-
	*Coscinodiscus grani*	MW435847	33/2	24	0	-
	*Coscinodiscus wailesi*	MW1228411	34/2	24	0	-
	*Melosira undulata*	NC_037728	34/2	24	0	-

*The 35 PCGs included (atp6, 8, 9; cob; cox1, 2, 3; nad1-7, 4L, 9, 11; rpl2, 5, 6, 14, 16; rps2, 3, 4, 7, 8, 10,11, 12, 13, 14, 19; tatA, and tatC) and three rRNAs included rnl, rns, and rrn5.*

Independent gene loss events have been identified in these mtDNAs. While the 5S rRNA gene (*rrn5*) was missing in the 52 of 68 mtDNAs, most other genes were missing in mtDNAs of only a few species. The gene *rps2* was found missing in eight mtDNAs, including four mtDNAs of species in Mediophyceae and three mtDNAs of species in Coscinodiscophyceae (Figure 4). The gene *tatA* was found missing in two mtDNAs, both of which were species in Coscinodiscophyceae. Other genes, including *rps7*, *rps10*, *rps12*, *rps13*, *rps19*, and *rpl5*, were found to be missing in single instances of these 68 mtDNAs, suggesting that these genes were likely disposable from the mtDNAs. Indeed, similarity searches of genes *rps2*, *rps13*, *rps19*, and *tatA* that were lost in mtDNAs in their corresponding nuclear genomes did not identify close homologs. In the class Mediophyceae, mtDNAs of *Lithodesmioides* sp., *Odontella regia*, and *Lithodesmium undulatum* each lost a single PCG *rps2*. In the class Coscinodiscophyceae, *Guinardia striata* mtDNA lost a single PCG *tatA*, while *Actinocyclus* sp. mtDNA lost four PCGs *rps2*, *rps13*, *rps19*, and *tatA*. In addition, *Coscinodiscus grani* mtDNA lost two PCGs *rps10* and *rpl5*, mtDNAs of *Coscinodiscus wailesi* and *Melosira undulata* each lost a single PCG *rps2.* In the class Bacillariophyceae, mtDNAs of three species each lost a single PCGs. *Synedra acus* mtDNA lost *rps2*, *Pseudo-nitzschia multiseries* mtDNA lost *rps7*, while *Halamphora calidilacuna* mtDNA lost *rps12*.

### Phylogenetic Positions of the Mitochondrial Genomes of the 15 Species

**FIGURE 5:**
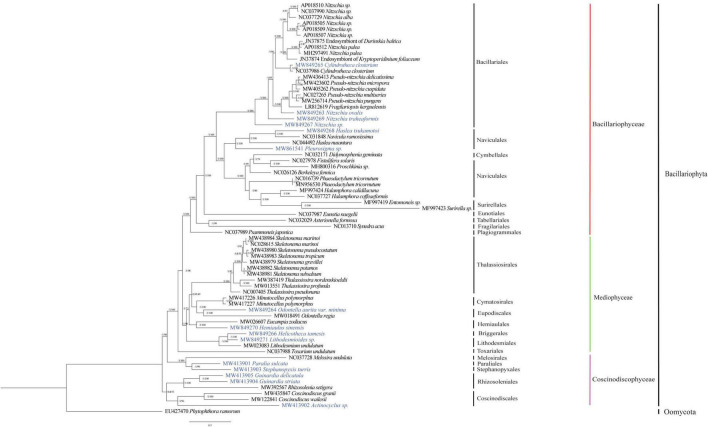
Maximum likelihood (ML) phylogenetic tree based on concatenated amino acid sequences encoded by 27 mitochondrial protein-coding genes (*atp6, 8, 9; cob; cox1, 2, 3; nad1-7, 4L, 9, 11; rpl2, 6, 14, 16; rps3, 4, 8, 11, 14; and tatC*). Mitochondrial protein-coding genes of *Phytophthora ramorum* were used as outgroup taxa. Numbers on the left and right side at the branches represent Bayesian posterior probabilities and bootstrap values. Branch lengths were proportional to the amount of sequence change, which are indicated by the scale bar below the trees.

To explore the evolutionary relationships between mtDNAs of 15 species constructed in this project and those of other diatom species reported previously, we constructed a phylogenetic tree using amino acid sequences of PCGs shared by these mtDNAs using maximum likelihood method. As expected, all diatom species generally fell into three clades, corresponding to three distinct classes Bacillariophyceae, Mediophyceae, and Coscinodiscophyceae (Figure 5). Mitochondrial genomes representing 39 species in the class Bacillariophyceae were classified into nine orders. Of the 15 mtDNAs constructed in this study, six mtDNAs belonged to the orders Bacillariales and Naviculales in this class. In Bacillariales, *Cylindrotheca closterium* (MW849265 of the strain CNS00381) clustered with another strain of *Cylindrotheca closterium* (NC_037986) as expected (Guillory et al., 2018). The mtDNAs of *Nitzschia ovalis*, *Nitzschia traheaformis*, and *Nitzschia* sp. did not cluster with mtDNAs of other species in the genus *Nitzschia*, suggesting that that the genus *Nitzschia* may actually represent multiple genera. In the order Naviculales, *Haslea tsukamotoi* was found to group more closely with *Navicula ramosissima* than with *Haslea nusantara* suggesting that that the genus *Navicula* and *Haslea* had more complex relationships (An et al., 2016; Prasetiya et al., 2019). And the mtDNA of *Pleurosigma* sp. was found to form an independent clade (Figure 5).

Twenty mtDNAs (including four mtDNAs constructed in this study) were found in the Mediophyceae clade, including species representing seven orders. *Odontella aurita var. minima* mtDNA was grouped with that of *Odontella regia* in the order Eupodiscales as expected (Wang et al., 2021). *Hemiaulus sinensis* mtDNA was clustered with that of *Eucampia zodiacus* (Zhang M. et al., 2021) in the order Hemiaulales. *Lithodesmioides* sp. mtDNA, which belonged to the order Lithodesmiales, was clustered more closely with the mtDNA of *Helicotheca tamesis* from another order Briggerales than with the mtDNA of *Lithodesmium undulatum* that belongs to the same order Lithodesmiales (Figure 5).

In the class Coscinodiscophyceae, nine mtDNAs (including five mtDNAs constructed in this study) representing five orders have been constructed. Mitochondrial genome of *Paralia sulcata*, which represented the first species of the order Paraliales, was clustered with the mtDNA of *Stephanopyxis turris*, which also represented the first species of the order Stephanopyxales. In the order Rhizosoleniales, mtDNAs of *Guinardia delicatula* and *Guinardia striata* from the same genus *Guinardia* formed a clade within the same order that also included *Rhizosolenia setigera*. Finally, mtDNA of *Actinocyclus* sp. was clustered with that of *Coscinodiscus granii* and *Coscinodiscus wailesii* (Figure 5).

Taken together, the 15 mtDNAs constructed in this study were grouped into 14 orders of diatom species. Notably, the mtDNAs of *Helicotheca tamesis*, *Paralia sulcata*, and *Stephanopyxis turris*, represented the first mtDNAs of the orders Briggerales, Paraliales, and Stephanopyxales, respectively, providing first reference mtDNAs for these orders.

**FIGURE 6:**
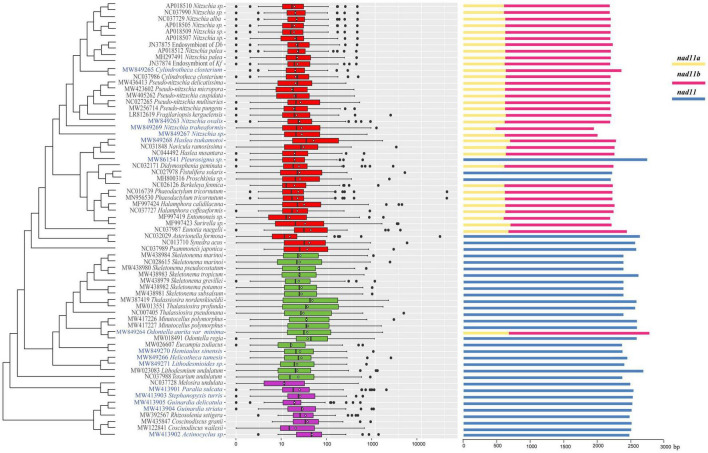
Boxplot of intergenic region length and the structure of nad11gene of 68 mtDNAs. On the left part, red boxes, green boxes, and purple boxes represented the three classes Bacillariophyceae, Mediophyceae, and Coscinodiscophyceae, respectively. The white point and black vertical line of every box were average and median of intergenic region length, respectively. The scale of intergenic region length is in a log10 units. On the right part, *nad11a* and *nad11b* were two unit caused by the break of the *nad11* gene.

We further explored the sizes of intergenic regions of mtDNAs in the context of the phylogenetic relationships (Figure 6). As expected, the intergenic regions of diatom mtDNAs were generally small, with only minor exceptions. Coding sequence of the gene *nad11* was split into two segments in the mtDNAs of many diatom species. The availability of 15 additional mtDNAs allowed us to systematically examine the evolutionary features of *nad11* (Figure 6). Among 39 mtDNAs of class Bacillariophyceae, *nad11* showed split model in 33 mtDNAs (Figure 6). The gene *nad11* was not split in the mtDNAs of six species including *Pleurosigma* sp., *Fistulifera solaris*, *Proschkinia* sp., *Psammoneis japonica*, *Asterionella Formosa*, and *Synedra acus*. In contrast, the gene *nad11* was not split in all mtDNAs of class Mediophyceae except *Odontella aurita var.minima*. In the class Coscinodiscophyceae, none of the *nad11* genes were split in the mtDNAs of all species, suggesting that the split of *nad11* was occurred multiple times in evolution.

### Synteny Analysis of Full-Length Mitochondrial Genomes

**FIGURE 7:**
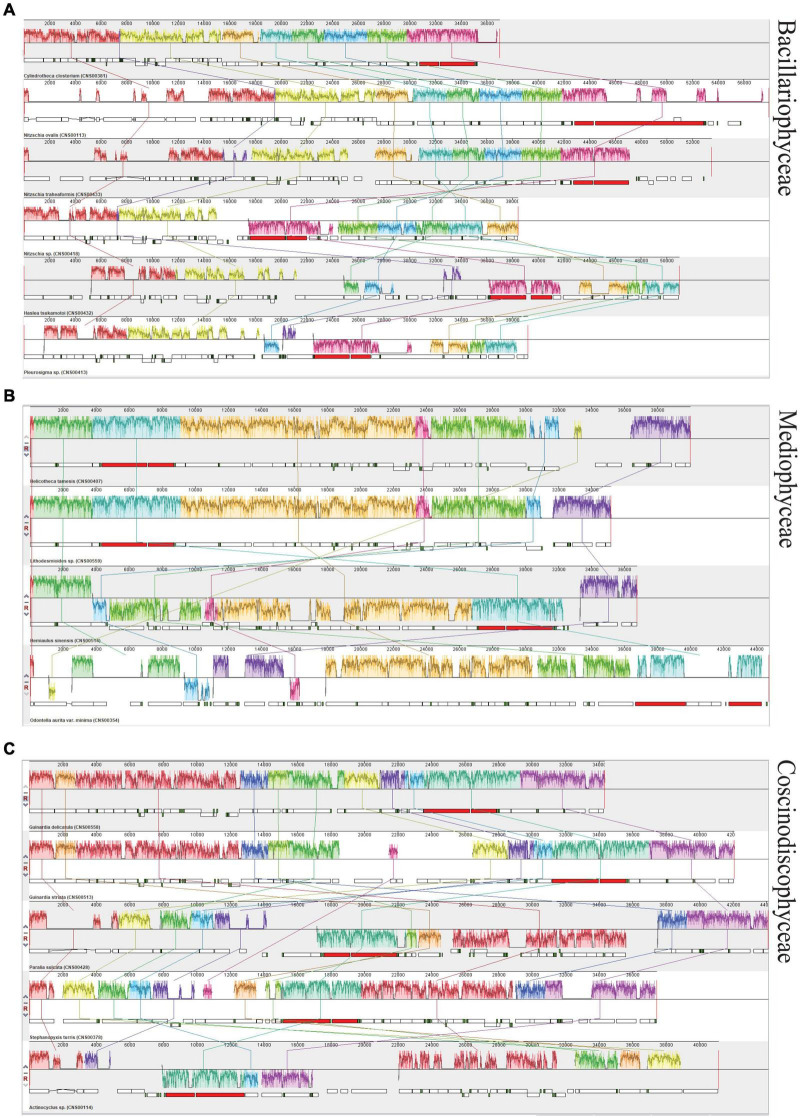
Synteny analysis of diatom mtDNAs constructed in this study. **(A)** Synteny relationships among six newly constructed mtDNAs of Bacillariophyceae based Mauve analysis. **(B)** Synteny relationships among four newly constructed mtDNAs of Mediophyceae. **(C)** Synteny relationships among five newly constructed mtDNAs of Coscinodiscophyceae. Each colored block indicates a synteny block among different mitochondrial genomes.

In the class Bacillariophyceae, six mtDNAs constructed in this study exhibited highly diverse gene arrangements (Figure 7A). Similar situation was also found in the classes Mediophyceae and Coscinodiscophyceae. Four mtDNAs constructed in this study that belonged to the class Mediophyceae and five mtDNAs constructed in this study that belonged to the class Coscinodiscophyceae also had a variety of rearrangements, respectively (Figures 7B,C).

**FIGURE 8:**
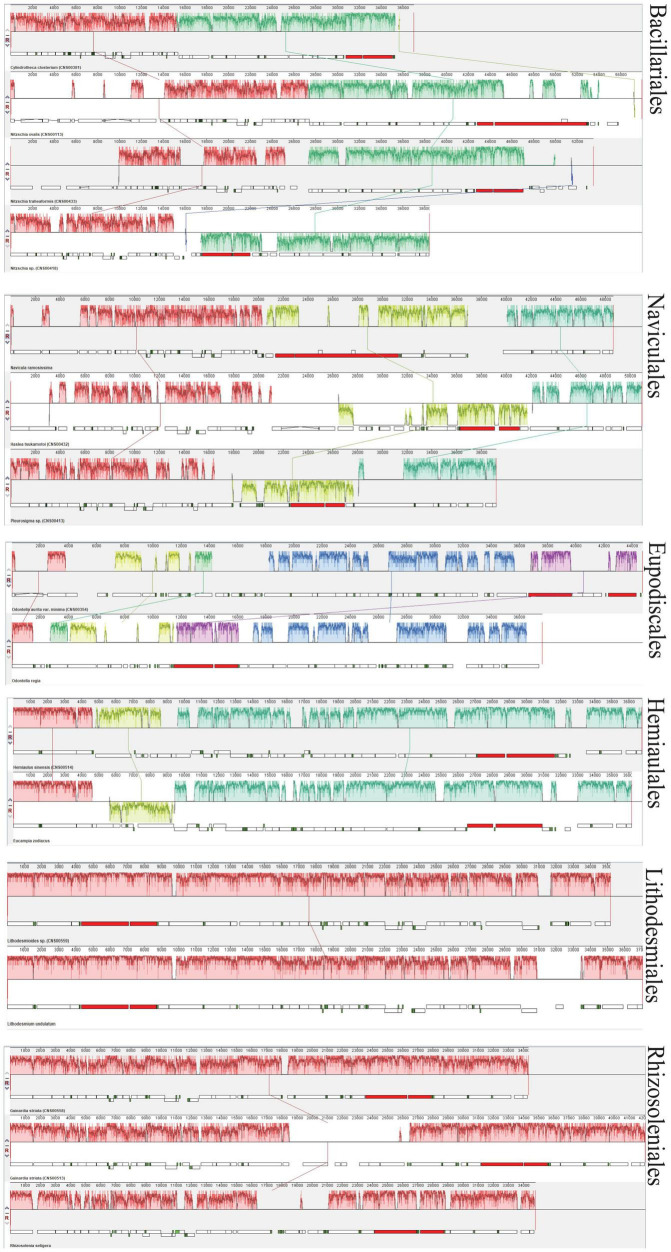
Synteny relationships using Mauve analysis for six orders of diatoms (Bacillariales, Naviculales, Eupodiscales, Hemiaulales, Lithodesmiales, and Rhizosoleniales) that included mtDNAs constructed in this study. Each colored block indicates a synteny block among different mitochondrial genomes.

**FIGURE 9:**
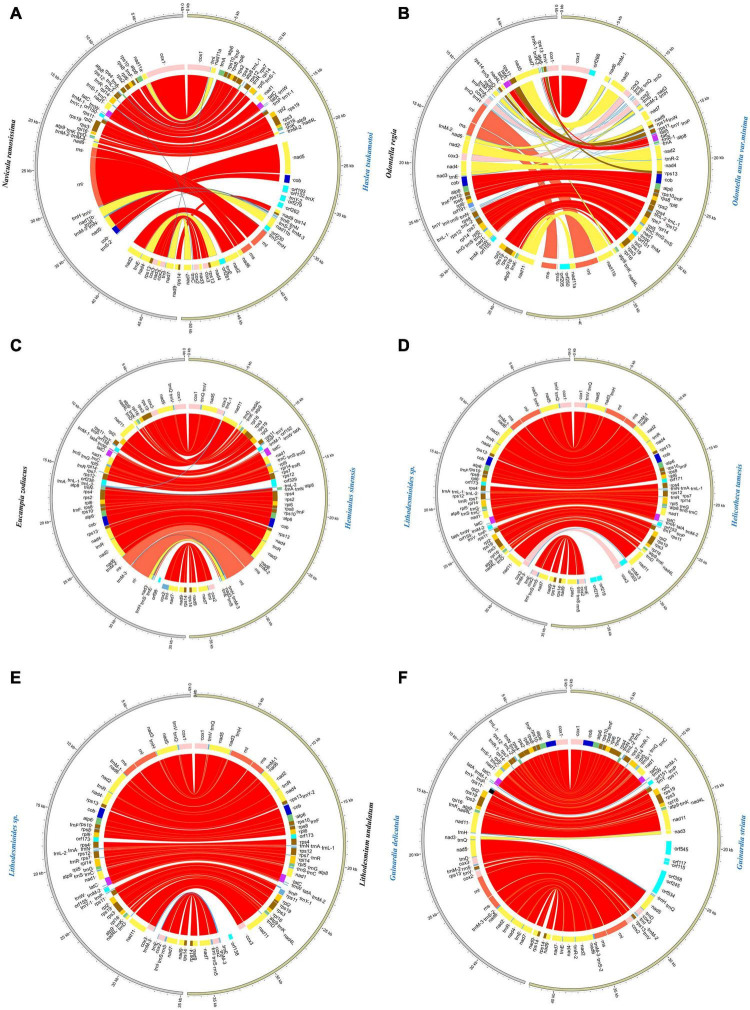
Pair-wise comparison of representative mtDNAs. **(A)** The comparative analysis of *Haslea tsukamotoi* and *Navicula ramosissima* mtDNAs. **(B)** The comparative analysis of *Odontella aurita var. minima* and *Odontella regia* mtDNAs. **(C)** The comparative analysis of *Hemiaulus sinensis* and *Eucampia zodiacus* mtDNAs. **(D)** The comparative analysis of *Helicotheca tamesis* and *Lithodesmioides* sp. mtDNAs. **(E)** The comparative analysis of *Lithodesmium undulatum* and *Lithodesmioides* sp. mtDNAs. **(F)** The comparative analysis of *Guinardia striata* and *Guinardia delicatula* mtDNAs. The assignment of genes into different functional groups is indicated by colors. The conserved back-to-back arrangement of genes was all shown with same color.

For exploring the degree of conservation of the mtDNAs at the order level, we conducted a series of synteny analysis in six orders that contained mtDNAs constructed in this study (Figure 8). The mtDNAs of the same order showed more conservation with fewer genome rearrangement events than that of different orders. For example, the mtDNAs of two species *Lithodesmioides* sp. and *Lithodesmium undulatum* of the order Lithodesmiales showed nearly identical genome organization. Gene-level analysis revealed that gene translocations and/or inversion in the block of *cox2-trnE-trnM-3* appeared in *Lithodesmioides* sp. and *Lithodesmium undulatum*, *Helicotheca tamesis*, and *Lithodesmioides* sp. (Figures 9D,E). The mtDNAs of three species *Guinardia delicatula*, *Guinardia striata*, and *Rhizosolenia setigera* of the order Rhizosoleniales were also highly syntonic with no rearrangements. Gene-level analysis of *Guinardia striata* and *Guinardia delicatula* mtDNAs showed that most genes arrangements were conserved except for the translocations of *trnM-1*, *trnH*, and *nad3* (Figure 9F).

In other orders, some genome rearrangements were identified. In the order Bacillariales, a major genome arrangement event (i.e., an inversion) was identified between the mtDNA of *Nitzschia* sp. and the mtDNAs of three other species in this order (*Cylindrotheca closterium*, *Nitzschia ovalis*, and *Nitzschia traheaformis*) (Figure 8). In the order Naviculales, a major genome rearrangement event (i.e., an inversion) was also identified between the mtDNA of *Navicula ramosissima* and mtDNAs of two other species (*Haslea tsukamotoi* and *Pleurosigma* sp.) (Figure 8). Further gene level analysis of *Navicula ramosissima* and *Haslea tsukamotoi* mtDNAs revealed multiple translocation events including *trnI*, *nad11a*, *trnA*, *trnR*, *trnN*, *trnS*, *trnM-3*, *nad11b*, *trnC*, *nad4*, and *nad7* and the inversion of a gene block containing *rnl*, *rns*, and *nad6* (Figure 9A). In the order Eupodiscales, multiple translocation events were identified between the mtDNAs of two species of a same genus, *Odontella aurita var.minima* and *Odontella regia*, breaking the genes in these two genomes into five gene blocks (Figures 8, 9B). Notably, all genes on the mtDNAs of both *Odontella aurita var. minima* and *Odontella regia* were located on a single strand (Figure 8). In the order Hemiaulales, a major inversion event was identified between two species, *Hemiaulus sinensis* and *Eucampia zodiacus*. The gene arrangements of *Hemiaulus sinensis* and *Eucampia zodiacus* mtDNAs were almost consistent with seven gene rearrangements including *trnL-1*, *trnL-2*, *rns*, *rnl*, *trnM-3*, *trnH*, and *nad3* (Figure 9C).

### Divergence Time Estimation for the Diatom Species

**FIGURE 10:**
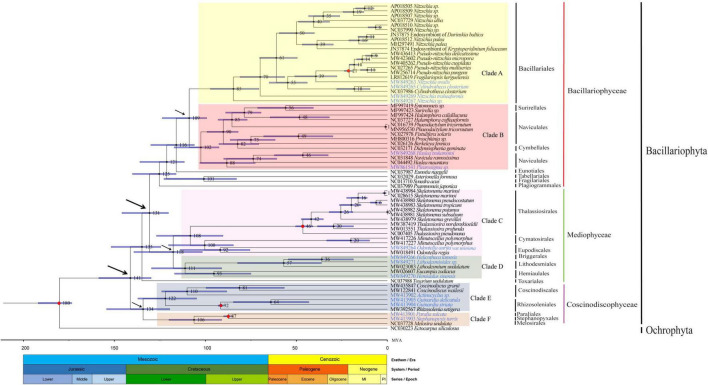
Time-calibrated phylogeny of 68 mtDNAs based on 27 shared PCGs in the mtDNAs of three classes of Bacillariophyta and the outgroup (*Ectocarpus siliculosus*). The red dots represent calibration point and the 95% highest posterior density interval for node ages are shown with translucent blue bars.

The availability of mtDNAs allowed us to explore the divergence time between diatom species, the speciation of which originated in Jurassic period (95% HPD: 173–192 Mya) (Lewitus et al., 2018) (Figure 10). The divergence between the classes Coscinodiscophyceae and Mediophyceae occurred 141 million years ago (Mya, 95% HPD: 159–130 Mya), while the divergence between the classes Mediophyceae and Bacillariophyceae occurred in 131 Mya (95% HPD: 146–120 Mya). The class Bacillariophyceae were represented by two major clades, including Clade A containing a single order Bacillariales, and Clade B containing three orders (e.g., Surirellale, Naviculales, and Cymbellales). The divergence between of the Clade A and Clade B occurred 109 Mya (95% HPD: 124–99 Mya). In Clade B, the order Naviculales was divided into two subclades, suggesting that this order should be divided into two independent orders.

The class Mediophyceae contained Clade C that consisted of the orders Thalassiosirales, Cymatosirales, and Eupodiscales, and Clade D that consisted of the orders Hemiaulales, Lithodesmiales, and Briggerales. In Clade C, the divergence between the order Thalassiosirales and the orders Cymatosirales and Eupodiscales occurred 108 Mya (95% HPD: 127–90 Mya), followed by the divergence of the orders Cymatosirales and Eupodiscales at 100 Mya (95% HPD: 120–85 Mya). In Clade D, the divergence between the orders Briggerales, Lithodesmiales, and Hemiaulales occurred at 111 Mya (95% HPD: 130–91 Mya), (95% HPD: 130–91 Mya), followed by the divergence of the order Briggerales from the order Lithodesmiales at 36 Mya (95% HPD: 55–19 Mya).

The class Coscinodiscophyceae contained Clade E that consisted of the orders Coscinodiscales and Rhizosoleniales, and Clade F that consisted of the orders Paraliales, Stephanopyxales, and Melosirales. These two clades diverged at 134 Mya (95% HPD: 154–120 Mya). In Clade E, the divergence between the orders Coscinodiscales and Rhizosoleniales occurred 122 Mya (95% HPD: 137–105 Mya). In clade F, the divergence between the orders Paraliales and Stephanopyxales from Melosirales occurred 106 Mya (95% HPD: 120–91 Mya).

## Discussion

### Diatom Biodiversity and Speciation

Although diatoms have been regarded to be enormously species-rich with 100,000–200,000 species, only 12,000 species have been described based on their morphological features with some molecular support (Mann and Vanormelingen, 2013). As a consequence, the quantitative understanding of diatom biodiversity has been limited. The recent development of remarkable genomics technologies and bioinformatics methods have made it possible to systematically construct mtDNAs with high throughput and low cost. Thus, organelle genomes including mtDNAs have been proposed to be used as “super barcodes” that provide higher resolution for distinguishing. As a demonstration of this idea, we have constructed 15 mtDNAs for diatom species isolated from the Jiaozhou Bay, which is an epitome of China’s offshore ecosystem (Liu et al., 2004). This research has demonstrated that systematic construction of diatom mtDNAs is an effective and fruitful approach for exploring diatom biodiversity and speciation.

### Gene Loss and Gene Duplication

Ribosomal PCGs of the mtDNAs have been lost more often than other genes that were related to respiration including cytochrome b (*cob*) and cytochrome oxidase subunit 1 (*cox1*) (Adamsa and Palmer, 2003). Among 68 diatom mtDNAs, the absence of specific ribosomal PCGs occurred sporadically and independently in only a few mtDNAs instead of widespread loss, which indicated that the function of the missing genes was still needed and may be directly replaced by some preexisting genes just like the gene substitution in yeast and plant mtDNAs (Karlberg et al., 2000; Adams et al., 2002). The loss of *tatA* from mtDNAs was first discovered in the diatom species *Guinardia striata* and *Actinocyclus* sp., and *rrn5* was found to be absent from most diatom mtDNAs (Oudot-Le Secq et al., 2001).

Gene duplication and subsequent divergence played a vital role in evolution by providing genetic material for novel functions (Hoshino et al., 2001; Taylor et al., 2001). In this study, the duplication and subsequent divergence of *cox1* gene in the mtDNAs of *Nitzschia traheaformis* and *Haslea tsukamotoi* was found for the first time in diatom mtDNAs. Two *cox2* genes were previously found in mtDNA of rapeseed (*Brassica napus* L.) (Handa, 2003). Further study of more mtDNAs will help us to learn the origin of gene duplication and explore the role gene duplication plays in the evolution of diatom mtDNAs, which will in turn facilitate research on diatom biodiversity and speciation.

### Phylogenetic Relationships Between Diatoms

Many studies had been conducted to explore the evolutionary relationships among diatom mtDNAs. For instance, the class Coscinodiscophyceae was found to be non-monophyletic according to the comparative analysis of 17 mtDNAs of diatoms (Pogoda et al., 2019), while the class Mediophyceae was found to be non-monophyletic and was a sister group of the class Bacilariophyceae according to the analysis of 35 mtDNAs of diatoms (Wang et al., 2021). In this study, the phylogenetic analysis of 68 mtDNAs covering 20 orders indicated that Mediophyceae and Coscinodiscophyceae were non-monophyletic. In the order Bacillariales, the taxonomy of species-rich *Nitzschia* was found to be unstable (Trobajo et al., 2013), which may contribute to the complicated genetic evolution relationships in genus *Nitzschia*. The published study had proposed transferring *Haslea tsukamotoi* to *Navicula* according to morphological data and molecular marker analysis (Li et al., 2017). That *Haslea tsukamotoi* was closer to *Navicula ramosissima* than *Haslea nusantara* in our tree also underscored this point. Similar situation was found in the order Thalassiosirales in which *Skeletonema* species was found to be closer to species of the genus *Thalassiosira* rather than *Thalassiosira pseudonana*. *T. pseudonana* has been proposed to be more appropriately classified as *Cyclotella nana* on the basis of the phylogenetic analyses of morphological and molecular datasets (Alverson et al., 2011). More mtDNAs from different orders are needed to facilitate understanding the complex evolutionary relationships between pairs of orders including Naviculales and Cymbellales, Briggerales and Lithodesmiales, and Paraliales and Stephanopyxales.

The sizes of intergenic regions usually correlated linearly with the genome size (Watzlowik et al., 2021). The largest differences were due to repeat sequences. The longest intergenic region was found in *Phaeodactylum tricornutum* and *Asterionella formosa* which contained 35 and 25 kb-long repeats, respectively (Secq and Green, 2011; Villain et al., 2017). The *nad11* split coding region made *nad11* gene undergo fission into two separate submits with its own start and stop codons, respectively and corresponds to the iron-sulfur binding (*nad11a*) and molybdopterin-binding domains (*nad11b*) (Guillory et al., 2018). In distinct contrast to the balance of intergenic region average length, the presence of *nad11* split coding region was obviously different in the three classes of diatom, only existing in the class of Bacillariophyceae and Mediophyceae, which pointed to that the break of *nad11* gene was produced during the evolution of diatoms diversity instead of generating randomly. In the class Coscinodiscophyceae, none of the *nad11* genes were split in the mtDNAs of all species suggesting that the split model of *nad11* was the derived version, while the non-split model of *nad11* was the original version.

### Synteny Conservation

There were a variety of rearrangements in three classes, which pointed to that the mtDNA structure was diverse between species in the phylum of Bacillariophyta. Other comparative analyses of mtDNAs had also shown a number of gene rearrangements in the class Eustigmatophyceae and class Phaeophyceae of algae, respectively (Sevcikova et al., 2016; Liu et al., 2019b). Compared to mtDNAs of different orders in the phylum of Bacillariophyta, the mtDNAs within same orders were more conserved. Interestingly, the transcriptional direction of genes on mtDNAs showed substantial variations. While genes are found on both strands of most mtDNAs, genes of mtDNAs of the genus *Odontella* (Eupodiscales) were found to be located on the same strand. The functional implication of such genome arrangement was yet unknown, but may represent a more economic transcription mode, as suggested previously in *Ulva pertusa* (Liu et al., 2017). Obviously further studies are needed to explore how such genome arrangement was acquired by *Odontella* species during evolution, and the functional implication it has in the formation of diversity in *Odontella*. Aside from the structural similarity, mtDNAs of *Lithodesmioides* sp. was closer to *Helicotheca tamesis* than *Lithodesmium undulatum* in the phylogenetic tree, suggesting that the mtDNA sequences evolved faster than their structures within order Lithodesmiales.

### The Origin and Speciation of Diatom Diversity

Fossil evidence suggested that diatoms originated in the late Jurassic period and became more common in the mid-Cretaceous (Round et al., 1990; Sims et al., 2006; Lewitus et al., 2018; Nakov et al., 2018). Despite the reliability of fossil records, many diatom deposits have undoubtedly been lost through diagenesis, making it difficult to track evolutionary trajectory of diatoms. Comparative analysis of diatom mtDNAs could provide valuable complementary genetic insights into diatom evolution. In this study, we first used diatom mtDNAs as a “super barcode” to infer the time frame within which the diatoms originated and diversified. Analysis of fossil records revealed that Mediophyceae and Coscinodiscophyceae diatoms evolved before Bacillariophyceae diatoms (Round et al., 1990). In our study, diatom species of the class Coscinodiscophyceae was first established, followed by diatom species of the class Mediophyceae, and diatom species of the class Bacillariophyceae were the last to appear, which was also generally consistent with the molecular phylogenetic dating analysis using nuclear-encoded small-subunit ribosomal RNA (Sorhannus, 2007). Our study indicated that all three classes of diatom emerged 100 Mya during the Cretaceous period (Figure 10). During this time frame, diatoms played dominant role in the carbon cycle, when CO_2_ levels of atmosphere were about five times higher than they are today and O_2_ levels were rising (Falkowski et al., 2005; Armbrust, 2009). The evolution of the habitat of the class Bacillariophyceae had happened in approximately 120 Mya which reflected the propensity of diatoms to occupy and adapt to a variety of habitats, from marine to the freshwater (Falciatore et al., 2020). Our study also suggested that freshwater species of Bacillariophyceae had appeared in 121 Mya (95% HPD: 112–136 Mya) such as *Asterionella formosa*. Many species began to appear after the end of Cretaceous, especially those of the order Bacillariales and Thalassiosirales. The mass extinction about 65 Mya which caused loss of about 85% of all species except diatoms and provided ecological opportunities for the establishment of new species (Falkowski et al., 2004). Throughout the Cenozoic, the rising diversity of diatoms had been attributed to an expanded bioavailability of silica from increased weathering of silicate rock (Cermeno et al., 2015) and an influx of nutrient-rich seawater into the South Atlantic brought by the Antarctic Circumpolar Current (Barker, 2001; Scher and Martin, 2006).

## Conclusion

The availability of 15 mtDNAs grouped in ten orders of diatom species provided valuable reference sequences for further evolutionary studies. The 15 newly constructed mtDNAs display several new features of mtDNAs including the identification of first duplication events of the *cox1* gene. Further study could help understand the role they play in the evolution and diversity formation of diatoms. The three classes of diatom are separate from each other. The evolutionary relationships for diatom species from different orders were complex, with mtDNAs within orders showing higher similarity. Diatoms of the class Coscinodiscophyceae appeared first in evolution. All three classes emerged 100 Mya during the Cretaceous period, the diversity of diatom began to rise obviously after the mass extinction about 65 Mya and many diatom species were generated 50 Mya. With the construction of more mtDNAs of more diatom species, more insight will be gained into the diatom biodiversity and speciation.

## Data Availability Statement

The datasets presented in this study can be found in online repositories. The names of the repositories and accession numbers can be found below: https://www.ncbi.nlm.nih.gov/, MW413901; https://www.ncbi.nlm.nih.gov/, MW413902; https://www.ncbi.nlm.nih.gov/, MW413903; https://www.ncbi.nlm.nih.gov/, MW413904; https://www.ncbi.nlm.nih.gov/, MW413905; https://www.ncbi.nlm.nih.gov/, MW849263; https://www.ncbi.nlm.nih.gov/, MW849264; https://www.ncbi.nlm.nih.gov/, MW849265; https://www.ncbi.nlm.nih.gov/, MW849266; https://www.ncbi.nlm.nih.gov/, MW849267; https://www.ncbi.nlm.nih.gov/, MW849268; https://www.ncbi.nlm.nih.gov/, MW849269; https://www.ncbi.nlm.nih.gov/, MW849270; https://www.ncbi.nlm.nih.gov/, MW849271; https://www.ncbi.nlm.nih.gov/, MW861541; and https://www.ncbi.nlm.nih.gov/, PRJNA746723.

## Author Contributions

NC conceived of the project. YW, SL, JW, YY, YC, and ZZ organized the strain selection, cultivation, and genome sequencing. YW and QX organized the assembly, annotation, quality control, and analyzed the data with suggestions from others. YW and NC wrote the manuscript with inputs from others. All authors read and approved the final version of the manuscript.

## Conflict of Interest

The authors declare that the research was conducted in the absence of any commercial or financial relationships that could be construed as a potential conflict of interest.

## Publisher’s Note

All claims expressed in this article are solely those of the authors and do not necessarily represent those of their affiliated organizations, or those of the publisher, the editors and the reviewers. Any product that may be evaluated in this article, or claim that may be made by its manufacturer, is not guaranteed or endorsed by the publisher.

## Footnotes

1
https://github.com/BFL-lab/Mfannot

2
https://www.ncbi.nlm.nih.gov/orffinder/

3
https://www.ncbi.nlm.nih.gov/guide/data-software/
