# Supplementary material for: Diatom Biodiversity and Speciation Revealed by Comparative Analysis of Mitochondrial Genomes
Source: Front Plant Sci. 2022 Mar 24;13:749982. doi: 10.3389/fpls.2022.749982 (PMC8987724; doi:10.3389/fpls.2022.749982)
Supplement: Supplementary file 1 [file Table_1.DOCX]

| calibration point | Mya | reference |
| --- | --- | --- |
| *Ectocarpus siliculosus* | 176-202 | (Matari and Blair 2014) |
| *Stephanopyxis turris* | 85-90 | (Sims et al. 2006) |
| *Rhizosolenia setigera* | 90-93 | (Damste et al. 2004) |
| Thalassiosirales | 40-50 | (Sims et al. 2006) |
| *Fragilariopsis kerguelensis* | 5-22 | (Sims et al. 2006) |

Damste JSS, Muyzer G, Abbas B, Rampen SW, Masse G, Allard WG, Belt ST, Robert JM, Rowland SJ, Moldowan JM, Barbanti SM, Fago FJ, Denisevich P, Dahl J, Trindade LAF, Schouten S (2004) The rise of the rhizosolenid diatoms. Science 304 (5670):584-587

Matari NH, Blair JE (2014) A multilocus timescale for oomycete evolution estimated under three distinct molecular clock models. Bmc Evolutionary Biology 14

Sims PA, Mann DG, Medlin LK (2006) Evolution of the diatoms: insights from fossil, biological and molecular data. Phycologia 45 (4):361-402
